# Looking inside the lab: a systematic literature review of economic experiments in health service provision

**DOI:** 10.1007/s10198-023-01662-y

**Published:** 2024-01-11

**Authors:** Massimo Finocchiaro Castro, Calogero Guccio, Domenica Romeo

**Affiliations:** 1 Department of Economics and Business, University of Catania, Corso Italia 55, 95123 Catania, Italy; 2 Department of Law, Economics and Humanities, Mediterranean University of Reggio Calabria, Reggio Calabria, Italy; 3 Health Econometrics and Data Group, University of York, York, UK; 4 Institute for Corruption Studies, Illinois State University, Normal, USA

**Keywords:** Laboratory experiments, Supply of health services, Systematic literature review, Physicians’ behavior, Payment systems, Health policy, C91, D90, I12

## Abstract

Experimental economics is, nowadays, a well-established approach to investigate agents’ behavior under economic incentives. In the last decade, a fast-growing number of studies have focused on the application of experimental methodology to health policy issues. The results of that stream of literature have been intriguing and strongly policy oriented. However, those findings are scattered between different health-related topics, making it difficult to grasp the overall state-of-the-art. Hence, to make the main contributions understandable at a glance, we conduct a systematic literature review of laboratory experiments on the supply of health services. Of the 1248 articles retrieved from 2011, 56 articles published in peer-review journals have met our inclusion criteria. Thus, we have described the experimental designs of each of the selected papers and we have classified them according to their main area of interest.

## Introduction

In the last decades, laboratory and field experiments have been designed to test theoretical models in many economic areas [23, 24], such as bargaining [77], auctions [52], public good provision [93] and finance [28]. More recently, economic experiments have proven to be a useful tool to test individual and organizational decision-making process related to health and healthcare [34]. In fact, controlled environments, such as experimental laboratory, allow to test *ex ante* the effects of health policy changes, like the introduction of new financial incentive schemes to physicians and minimize confounding effects when looking for causality nexus between variables. Such desirable feature becomes extremely relevant in health economics where agent’s behavior could affect individual wellness, has legal consequences and is ethically sensitive.

The merit of experimental methods led to a fast-growing literature in health economics, addressing several topics: risk and time preferences (e.g., [33], health insurance choices (e.g., [50]), providers’ incentives (e.g., [27]), altruism (e.g., [62]), competition (e.g., [12–14]), professional norms (e.g., [56]), malpractice (e.g., [30]), medicines price policies (e.g., [90]). Also, the design of experiments in health economics may vary in several dimensions [34] like, for instance, the wording of instructions (neutral vs. health-related) and the type of participants (students, medical students, or physicians) joining the experimental sessions [35, 53, 86]. Therefore, a comprehensive, systematic, and reader-oriented review of experimental health economics may be of help to guide scholars through this new stream of experiments. We believe our work contributes to fill this gap in the literature.

Also, we acknowledge that other scholars have previously reviewed some of the existing literature, though with different purposes. Galizzi and Wiesen [34] critically discuss the state-of-the-art, explaining the methodologies, debating potential areas of application of experiments to health, and thus suggesting scopes for further research. Also, Vlaev et al. [84] summarize the available literature on the use of financial incentives to change health behaviors. However, whereas the former could be classified as a methodological paper, rather than a review, the latter focuses on a very specific subject, lacking the comprehensiveness which we aim to achieve with our work. Finally, in a special issue of the Journal of Economic Behavior and Organization, Cox et al. [20–22] provide an overview of laboratory experiments in four different topics of healthcare research (i.e., clinical decision support, physicians’ incentives, healthcare systems and insurance, healthcare delivery, and public health), emphasizing in the conclusions all the strengths of experimental methods.

Thus, there is a lack of a comprehensive collection of the main contributions and their most relevant features from the supply side perspective. For this reason, we conduct a systematic literature review of the articles published in peer-reviewed journals from 2011, examining laboratory experiments in health economics focused on the supply of health services. The initial bibliographic metadata is drawn from the SCOPUS database. Of the 1248 articles retrieved from 2011, 56 articles have met our quite selective inclusion criteria, which restrict the attention to laboratory or online (hypothetical lab) experiments and to the experiments whose data have been gathered by merging laboratory and artefactual field experiments or combining lab/online experiments and non-experimental methods. For the sake of consistency, when the study results from the combination of a lab and a field experiment, we discuss only the aspects emerging from the former. Similarly, when lab or online experiments are merged with non-experimental methods (e.g., surveys or discrete-choice experiments), we look only at the experimental data. In fact, laboratory experiments can complement field experiments and non-experimental methods [34] and may serve as a ‘wind tunnel’, before really implementing a policy change or running a large-scale study [20–22]. According to Harrison and List [45], laboratory experiments are among the most appropriate methods for a counterfactual analysis (especially in the health field), since they allow for the identification of a control group, through randomization. In a nutshell, they define the gold standards for the environmental control [19]. Online experiments represent the main alternative to experiments run into the lab (especially under COVID-19 restrictions), given their lower costs to compensate for participants’ opportunity costs and the possibility to recruit large samples of participants which allow to perform high-powered tests^1^ and are more representative of the general population [44]. Differently, according to Charness et al. [19], field experiments are often infeasible, since they require sufficient variation, randomization, and the need to make the experiment ‘invisible’ to participants. Another drawback is the impossibility of replicating the experiment, which represents instead the main property of lab experiments [19]. Thus, we have decided to exclude field experiments, which do not allow for the level of experimental control that is critical for their internal validity. We have also excluded discrete-choice [25], control trials and quasi-experiments based on hypothetical decisions (stated preferences) only, since the incentive structure, together with the experimental control are fundamental aspects of experimental methods in economics (see [44]). Looking at the different areas of interest, we have detected one main macro-category topic, payment schemes, which covers a large portion of our dataset, although other research topics such as health insurance, competition and risk preferences will be discussed too.

The remainder of the paper is organized as follows. “Background” presents our background and the method applied in the systematic literature review, showing some preliminary results on bibliographic “metadata”; “Basic summary of the sampled publications” discusses the basic summary of the selected papers; “Review” describes the selected papers distinguished by topic. Finally, “Concluding remarks” provides some concluding remarks.

## Background

### Literature review method

In this section, we outline the method and selection criteria used to review the literature. First, we elaborate upon our selection criteria regarding the lab experimental approach in the field of health economics and the supply side perspective.

**Fig. 1:**
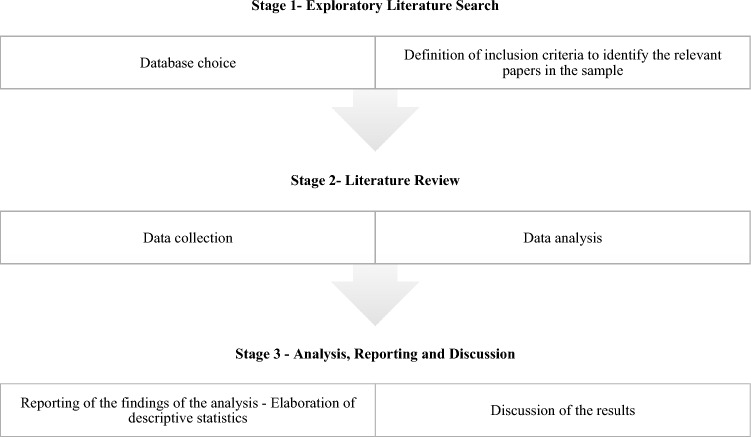
Main steps of the literature search and identification of studies. *Source:* our elaboration

According to Greenhalgh [41], a systematic review is nothing but an overview of primary studies which explicitly defines objectives, materials, and methods and has been conducted following an explicit and reproducible methodology. There are three main advantages for writing a systematic review: to summarize the existing evidence concerning a given topic, to detect any gap which leaves space for future research; to provide a framework which helps to locate new research activities in appropriate positions [57]. Although it shares some peculiarities with a traditional literature review, a systematic one must be looked at as a self-contained research project, which investigates a clearly defined issue [26]. Differently from systematic reviews, narrative reviews do not indicate neither the databases and the methodologies followed to perform the review, nor the inclusion criteria used to extract the dataset, thus preventing other authors from replicating the study [78]. Hence, we opt for a systematic review built in three main steps. First, we select the database to be investigated (for instance Scopus, Web of Science, PubMed, Google Scholar, etc.) and, by looking at the papers, we detect the keywords which allow us to build our search string. Throughout this stage, we select the papers to be analyzed, by defining our inclusion/exclusion criteria. In the second stage, we provide a descriptive and content analysis of the papers included in our sample. Finally, we focus on each of the selected papers, summarizing its contents and comparing different experimental settings and findings. Figure 1 reports the main steps of the literature search and identification of studies.

Several bibliographic databases, containing articles in peer-review journals and other types of publications, could potentially represent data sources to carry on a systematic review (for instance, Google Scholar, Web of Science (incl. MEDLINE), Scopus, EconLit, etc.). One relevant perspective to embrace, when choosing the most appropriate database, is whether it is endowed with a classification system that leads to the balancing of two conflicting goals: (1) to gather a wide coverage of the most suitable outlets where to publish papers focusing on our topic; 2) to allow to differentiate among publication subjects (e.g., [18]). Following the approach of Robinson and Botzen [76], we opt for the SCOPUS database in conjunction with Google Scholar, applying a parallel check through snowballing. Indeed, SCOPUS database spans from the general field of health to more specialized fields of health economics and experimental economics, offering a quite accurate definition of the subject areas and a good coverage of citation data in scholar journals. Thus, we are confident that SCOPUS database covers an extremely large proportion of the different experimental approaches used in health economics. We will explain below how we have managed to be reasonably confident that our sample of papers is as inclusive as possible of the literature that meets our inclusion criteria.

### Data collection

Once chosen the database, our systematic literature review process moves to data collection. We ran a database search to inform this review in July 2023.^2^ As previously explained, we limit our research to SCOPUS using a search string which includes the words lab, experiment, physician, and economic.^3^

To guarantee the quality of the selected works, we consider articles written in English language and published in peer-review journals from January 2011 onwards. Hence, we have found 1232 papers focusing on broad selection of topics and subjects.

The selection criteria are based on types of studies, types of experimental approaches, and types of topics. For a study to be included, it must deal with health economics topics, adopt the experimental methodology, look at the supply side, and be a laboratory experiment or an online experiment. To be crystal clear in defining our inclusion rule, we have considered eligible only papers in which subjects in the lab, and eventually merged with artefactual field sessions, or over the Internet, have been asked to provide health care services under different economic incentives. In the case of papers whose dataset is obtained by merging different types of methods, we have included only the results coming from laboratory or online sessions. Consequently, we have considered all other settings ineligible. Thus, we have excluded all those papers not related to health economics issues and those not applying the experimental methodology,^4^ at least mainly, in a controlled setting. Furthermore, from the health economics experimental papers, we have excluded all the experiments run in the field or natural experiments^5^ as well as discrete-choice experiments, control random trials and quasi-experiments, those based on hypothetical choices where the economic incentives to participants have been missing and experiments on health-related behavior [75].^6^

After reading titles, abstracts, and keywords, we have excluded 1151 articles that have not met our inclusion criteria. The full texts of the remaining 65 articles have been read in parallel by two researchers and, in the case of disagreement, by a third one (the so-called benefit of the doubt rule). Only 43 papers have passed the final selection meeting our eligible criteria, mainly in terms of experimental settings.

**Fig. 2:**
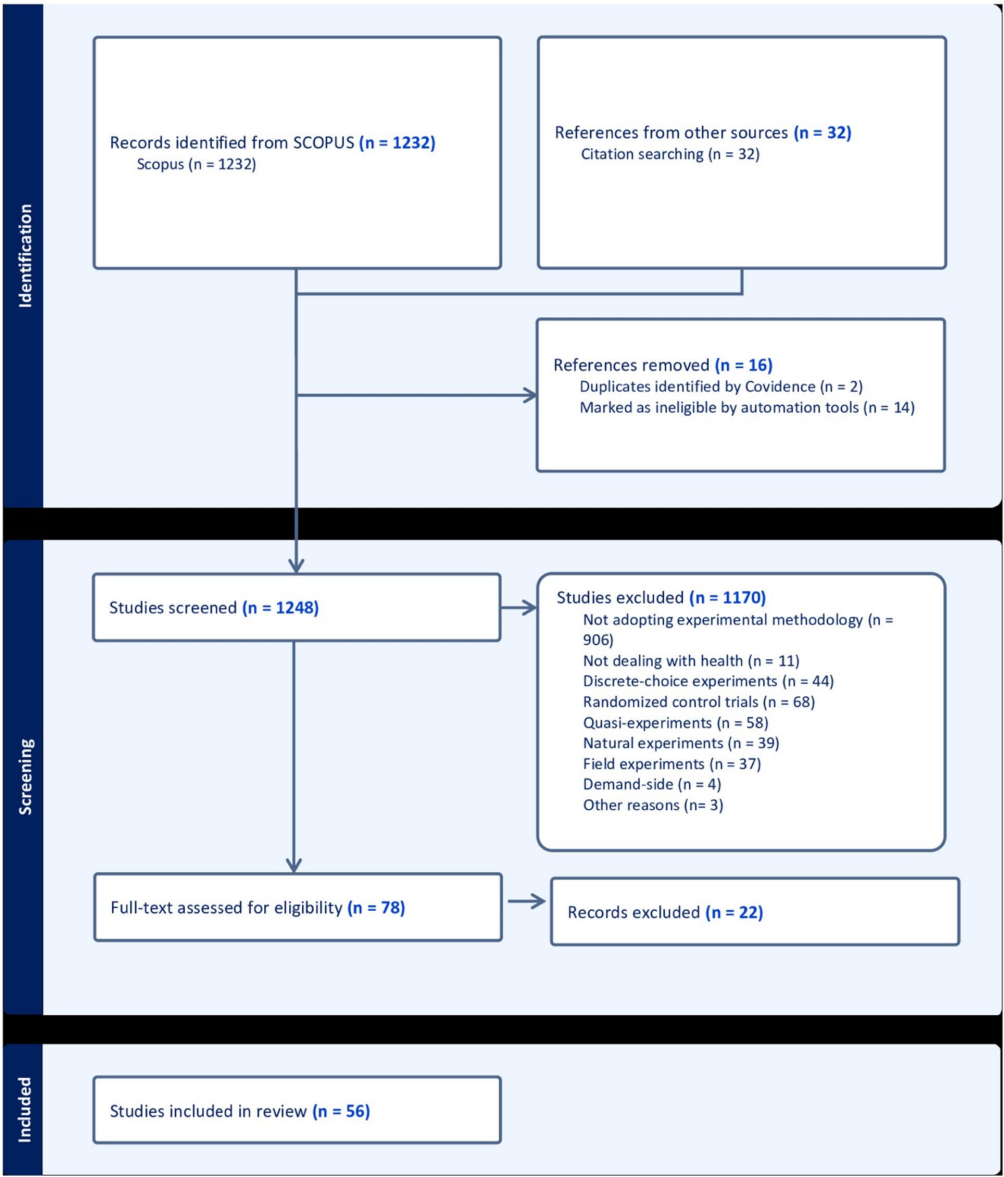
PRISMA flow diagram of the systematic literature review. Source: our elaboration

Since our search strategy and our choice of the SCOPUS database may have missed some important references on the topic, we apply a parallel check through snowballing, using the reference list in each paper and imputing the citations by a generic search engine^7^ (i.e., Google Scholar). Doing so, other 13 papers have been added, after looking at the references of the 43 selected papers, leading to a final sample of 56 papers. Figure 2 depicts the PRISMA flow diagram.

Although we have been careful in constructing our sample, we recognize that a limited number of studies may have been excluded because the search did not retrieve them or because checking by references and snowballing did not identify them. Nevertheless, we are reasonably confident that the included studies provide a complete and updated overview of the literature regarding the laboratory experimental approach in the field of health economics.

## Basic summary of the sampled publications

In this Section, we provide some descriptive statistics of the sampled publications. First, we show a synoptic table which collects all the reviewed papers, differentiating them by the sample size and the subject pool selected. Additionally, we discuss the trend of the papers by year, publishing journal and area of interest.

### Synoptic table

Table 1 lists the 56 papers of our sample and distinguishes them by the outlet, the topic, the sample size, whether the study was conducted over the Internet or resulted from a combination of experimental and non-experimental (i.e., extra-laboratory) methods, the employment of either medical students or physicians in the experiment and the number of citations. Numbers in parentheses in the last two columns indicate the specific number of that subject pool joining the experiment.

**Table 1:** Main features of the sample; *Source: our elaboration on Scopus database*

Code	Authors	Journal	Topic	Sample size in the lab	Medical Students in the lab	Physicians in the lab	Cited by
1	Ahlert et al. [1]	Health Economics Review	Distributive behavior	136	Yes (22)	No	17
2	Ahlert et al. [2]^a^	Social Choice and Welfare	Distributive behavior	326	Yes (107)	No	22
3	Angerer et al. [4]	Health Economics	Monitoring	424	No	No	5
4	Arrieta et al. [5]	Health Economics	Risk preferences	257	Yes (178)	No	22
5	Attema et al. [6]^b,c^	Journal of Health Economics	Altruism	878	Yes (733)	No	3
6	Bardey et al. [7]	Journal of Economic Behavior and Organization	Payment schemes	95	Yes (95)	No	1
7	Brendel et al. [9]	Health Economics	Medical service provision	174	Yes (13)	No	3
8	Brock et al. [10]^c^	Journal of Human Resources	Prosocial behavior	149	No	Yes (71)	2
9	Brosig-Koch et al. [11]^c^	Journal of Economic Behavior and Organization	Payment schemes	213	Yes (76)	No^a^	54
10	Brosig-Koch et al. [12]	Health Economics	Payment schemes	178	No	No	8
11	Brosig-Koch et al. [13]	Health Economics	Payment schemes	213	Yes (32)	No	66
12	Brosig-Koch et al. [14]	Health Economics	Payment schemes	185	Yes (28)	No	7
13	Brosig-Koch et al. [15]	Journal of Health Economics	Payment schemes	200	No	No	0
14	Brosig-Koch et al. [16]	Health Economics	Payment schemes	127	No	No	1
15	Cao and Liu [17]	IIE Transactions on Healthcare Systems Engineering	Diagnostic decisions	30	No	No	4
16	Cox et al. [20]	Journal of Economic Behavior and Organization	Payment schemes	209	Yes (209)	No	6
17	Cox et al. [21]	Journal of Economic Behavior and Organization	Discharge decisions	125	Yes (105)	Yes (20)	7
18	Di Guida et al. [27]	Health Economics	Payment schemes	38	Yes (38)	No	15
19	Finocchiaro Castro et al. [30]	Journal of Economic Behavior and Organization	Payment schemes	106	Yes (21)	Yes (4)	2
20	Ge and Godager [35]^c^	Journal of Choice Modelling	Market competition	136	No	No	6
21	Ge et al. [36]	Health Economics	Patient-regarding preferences	202	Yes (202)	No	1
22	Godager et al. [39]	Journal of Economic Behavior and Organization	Payment schemes	51	Yes (51)	No	15
23	Green [40]	Journal of Economic Behavior and Organization	Payment schemes	136	No	No	49
24	Greiner et al. [42]	Health Economics	Prescription behavior	300	No	No	6
25	Han et al. [43]	Health Economics	Hospital mergers	353	No	No	5
26	Hennig-Schmidt et al. [46]	Journal of Health Economics	Payment schemes	42	Yes (42)	No	144
27	Hennig-Schmidt and Wiesen [47]	Social Science and Medicine	Payment schemes	86	Yes (42)	No	53
28	Hennig-Schmidt et al. [48]	Health Economics	Payment schemes	98	Yes (51)	No	12
29	Herr and Normann [49]	Journal of Economic Behavior and Organization	Organ donation	192	Yes (21)	No	8
30	Huck et al. [50]	Journal of Economic Behavior and Organization	Medical insurance	336	No	No	29
31	Irvine et al. [51]	Social Science and Medicine	Payment schemes	109	Yes (36)	No	0
32	Kairies-Schwarz and Souček [53]^b^	International Journal of Environmental Research and Public Health	Payment schemes	56	Yes (40)	Yes (16)	2
33	Keser et al. [54]	Health Economics Review	Payment schemes	23	Yes (23)	No	2
34	Kessler and Roth [55]	Journal of Public Economics	Organ donation	608	No	No	18
35	Kesternich et al. [56]^b^	Journal of Public Economics	Professional norms	266	Yes (266)	No	47
36	Kolstad and Lindkvist [58]	Health Policy and Planning	Prosocial behavior	80	Yes (40)	No	21
37	Lagarde and Blaauw [59]	Social Science and Medicine	Payment schemes	132	Yes (132)	No	36
38	Laker et al. [60]	Production and Operations Management	Clinical decisions	24	No	yes (24)	34
39	Lee et al. [62]^b^	Plos one	Payment schemes	50	No	Yes (5)	1
40	Li et al. [63]^b^	Proceedings of the National Academy of Sciences	Social preferences	503	Yes (503)	No	28
41	Li et al. [65]^b^	Proceedings of the National Academy of Sciences	Social preferences	284	No	Yes (284)	5
42	Li et al. [66]	BMC Health Services Research	Payment schemes	210	Yes (210)	no	2
43	Martin-Lapoirie [67]	European Journal of Law and Economics	Teamwork	120	Yes (14)	No	1
44	Martinsson and Persson [68]	Theory and Decision	Payment schemes	130	No	No	13
45	Mimra et al. [69]	Journal of Economic Behavior and Organization	Second consultations	420	yes (8)	No	26
46	Oxholm et al. [70]	Applied Economics	Payment schemes	55	Yes (55)	No	5
47	Oxholm et al. [71]	Social Science and Medicine	Payment schemes	143	Yes (143)	No	7
48	Raptis et al. [73]^c^	Patient Preference and Adherence	Ambiguity aversion	73	No	Yes (73)	8
49	Reif et al. [74]	Journal of Environmental Research and Public Health	Payment schemes	126	No	Yes (21)	9
50	Saposnik et al. [80]^b,c^	Frontiers in neurology	Ambiguity aversion	96	No	Yes (96)	35
51	Wettstein and Boes [88]^b^	Health Economics Review	Payment schemes	404	No	No	6
52	Wettstein and Boes [90]^b^	Health policy	Payment schemes	269	No	No	21
53	Waibel and Wiesen [85]	European Economic Review	Payment schemes	252	Yes (50)	No	4
54	Wang et al. [86]	European Economic Review	Payment schemes	277	Yes (178)	Yes (99)	0
55	Zhang et al. [91]	BMC Health Services Research	Payment schemes	150	Yes (150)	No	2
56	Zhang et al. [92]^b,c^	BMC Health Services Research	Payment schemes	925	Yes (925)	No	0

^a^Questionnaire experiment, ^b^online experiment, ^c^combination of methods

^a^29 in the artefactual field

The average sample size of the selected papers is 210.36, ranging from a minimum of 23 to a maximum of 925 participants. Statistics sharply change when we take online experiments out of the calculation, with an average sample size of 191.83 (23–608), since studies conducted over the Internet allow for the recruitment of larger sample than those usually employed in the laboratory experiments. Fourteen studies have employed only nonmedical students. Physicians joined eleven experiments,^8^ four of which were run online, and four of which have employed medical students too. Restricting our attention to physicians, on average 64.81 of them take part in the experiments ranging from a minimum of 4 to a maximum of 99. Again, figures significantly change when we exclude online experiments, with an average sample size of 44.57.

The small number of studies including physicians is not surprising. In fact, running experiments with real physicians is not an easy task. As shown by Rahman et al. [72], in the context of clinical trial, physicians’ unwillingness to join sessions is due to several participation barriers such as lack of time, lack of incentives and recognition, communication troubles, absence of any research experience and in some circumstances ‘*a scientifically uninteresting research question*’, which makes them not involved at all. The same authors suggest the adoption of financial rewards to encourage doctors’ participation. However, it must be considered that physicians’ opportunity cost is very high, especially when compared with students’ opportunity cost, which is traditionally quite low. A possible solution could be to reach physicians via email and let them join the experiment online. Doing so, it would certainly raise the proportion of physicians accepting to participate, scheduling the sessions in a way to avoid interference with physicians’ working schedule. Allowing physicians to complete the experiment where and whenever they want without interfering with their work schedule would certainly make them more willing to contribute to the research and reduce their convenience costs. Certainly, this solution comes at the cost of a partial loss of the experimental control, which may result in the so-called observer effect (due to the absence of the experimenter supervising the sessions). However, many studies find little evidence of difference between behavior in the lab and outside [3, 61, 82]. Thus, the observer effect does not appear to significantly affect the experimental results.

Finally, looking at the citation frequency, Hennig-Schmidt et al. [46] stands out above all with 144 citations. This result is due to two main reasons. First, it is the oldest paper included in our review. Second, and most importantly, it laid the foundation of the artificial environment (i.e., the design) used to study the decision-making of physicians in terms of services provided to patients, given different payment structures. Then, Hennig-Schmidt et al. [46]’s design has been replicated with different subject pools (e.g., [11]) and different payment systems (e.g., [59]). Additionally, several variants of the standard design have been introduced, in order to test competition [16], altruism [91] and so on (see “The role of payment schemes”).

### Analysis of search results^9^

**Fig. 3:**
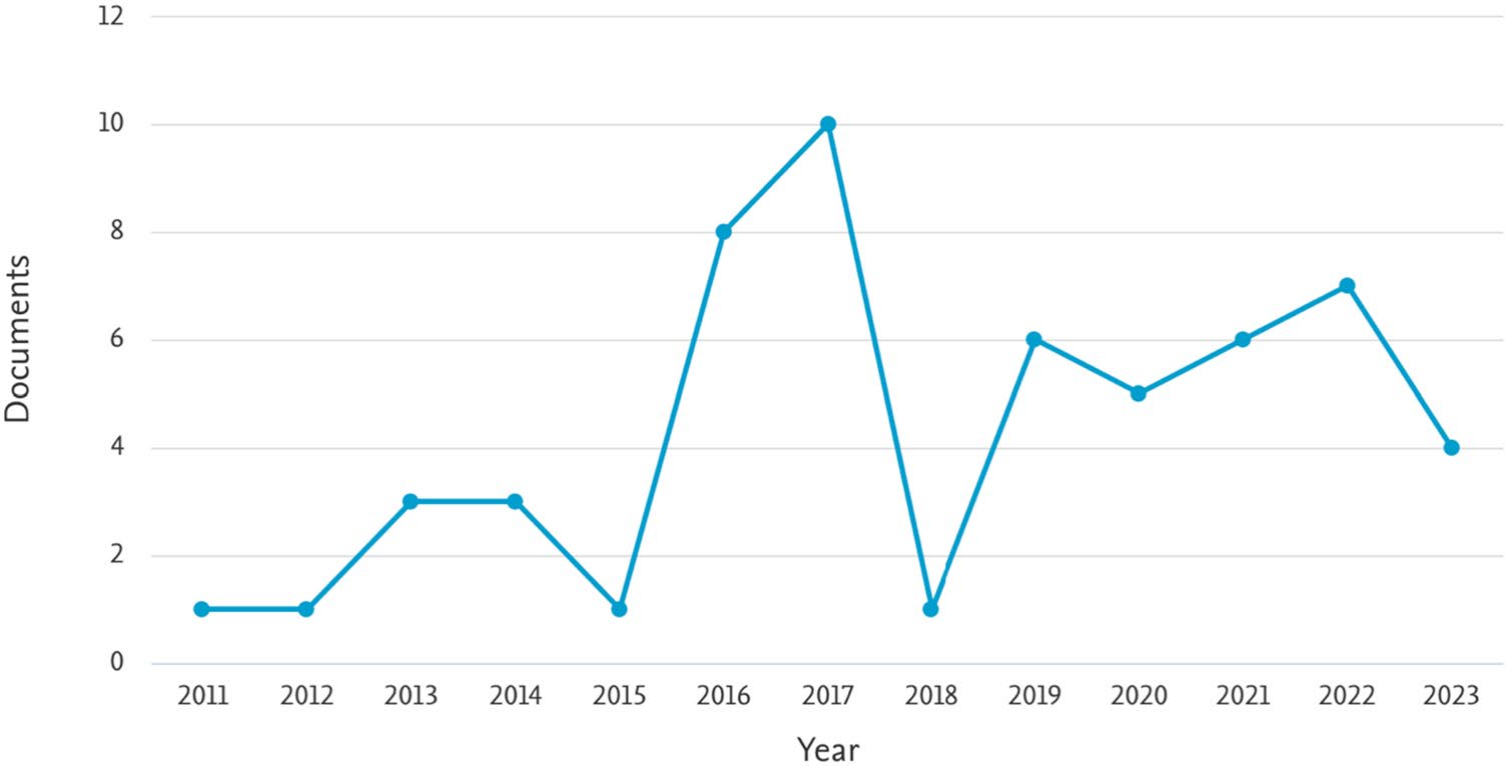
Documents by year. *Source*: our elaboration on Scopus database

Figure 3 shows the number of published papers by year.

The first paper was published in 2011, one paper in 2012, three papers each in 2013 and 2014, and then, one paper in 2015.^10^ In 2016–2017, we have witnessed two peaks with eight papers published in 2016 and ten in 2017. As shown by Cox et al. [20–22], in recent years, the pros of experimental methods applied to the healthcare have emerged, driving many authors to employ the behavioral approach to investigate many issues in this field. Finally, from 2018 up to the present year, we have observed a volatile pattern maybe due to the exclusion of field experiments from our review. However, we expect a downward trend in the 2-year period 2021–2023 due to the outbreak of COVID-19 in 2019–2020, which prevented experimenters from running sessions because of national restrictions. Such drop has been partially compensated by the use of online experiments (e.g., [90, 92]).

**Fig. 4:**
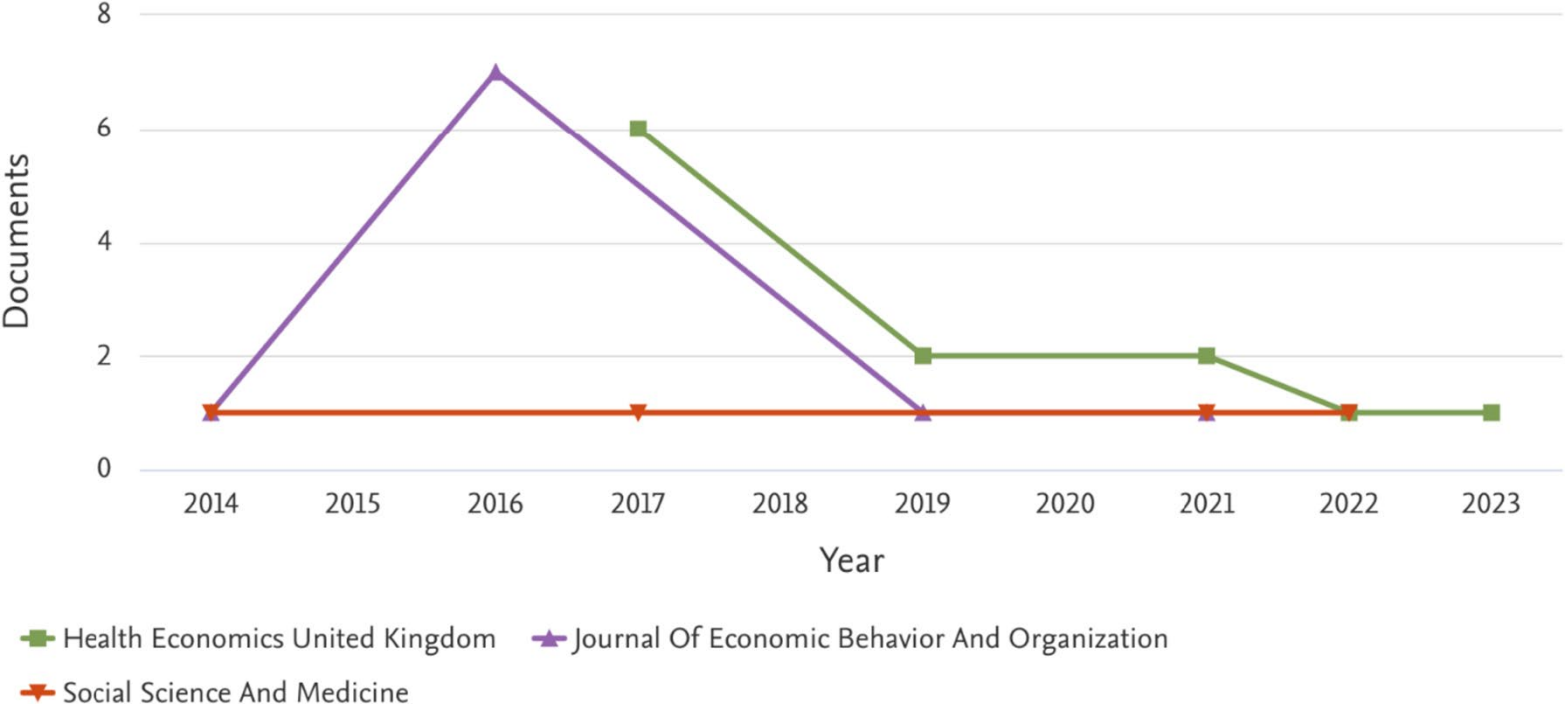
Documents per year by source. *Source*: our elaboration on Scopus database

Figure 4 reports the documents per year by source.

**Fig. 5:**
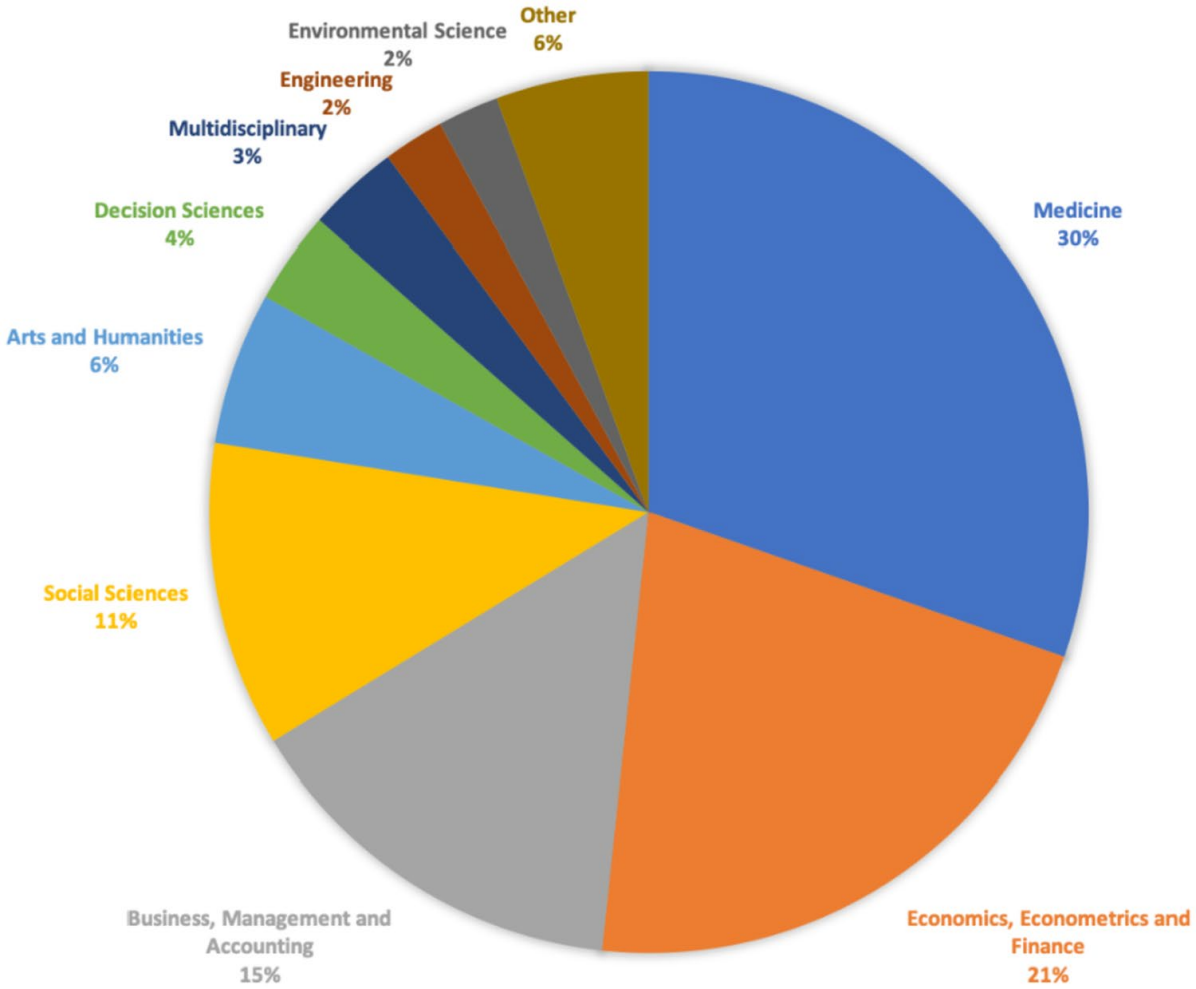
Documents by subject area. *Source*: our elaboration on Scopus database

We restricted the attention to the top three journals in terms of number of published papers: Health Economics (12 papers); Journal of Economic Behavior and Organization (10 papers); Social Science & Medicine (3 papers) accounting for 45% of the sampled papers (25 over 56). Figure 4 mirrors Fig. 3 to some extent, with two peaks in the two-year period 2016–2017 in correspondence of Journal of Economic Behavior and Organization and Health Economics, certainly due to the launches of the special issues ‘*Experimental and Behavioral Economics of Healthcare*’^11^ and ‘*Behavioural experiments in Health supplement*’,^12^ respectively. Finally, Fig. 5 differentiates documents by subject area.

As expected, 21% of the selected papers falls under the scope of Economics, Econometrics and Finance. 30% of the published studies belongs to Medicine, followed by Business, Management and Accounting (16%), Social Science (11%) and Arts and Humanities (6%). The breakdown by the remaining areas is almost equitable.

## Review

In the following paragraphs, we will first focus on the papers investigating the role of payment schemes on physicians’ decisions, accounting for 55.3% of our sample, and then, on those works not falling into any specific research topic group.

### The role of payment schemes

Measuring how physicians respond to payment schemes is the most common topic among experiments in health economics that look at the provision of healthcare services (i.e., supply side). In our systematic review, 31 articles out of 56 deal with physicians’ payment schemes. Table 2 summarizes the topic investigated and the main results of each paper.

**Table 2:** Studies in the sample that explore the role of payment schemes; *Source*: our elaboration

Authors	Topic	Results
Bardey et al. [7]	Personalized medicine and P4P, CAP and FFS	- Pay for performance (P4P) scheme incentives the adoption of personalized medicine compared to CAP and FFS - Information on personalized medicine improves providers’ performance regardless of the payment condition (though the effect is larger under FFS) - When information is costly, and once controlled for self-selection, personalized medicine increases the quality of care
Brosig-Koch et al. [11]	FFS and CAP	- Participants overtreat patients under FFS and undertreat them under CAP - Medical and nonmedical students differ in how strongly they respond to incentives. Medical students provide fewer services than nonmedical ones under FFS - The amount of services provided increases with patient’s severity of illness
Brosig-Koch et al. [12]	Competition between physicians and payment schemes (CAP and FFS)	- Competition reduces deviations from patient-optimal treatment, mitigating both overprovision in FFS and underprovision in CAP - Competition effects depend on patient characteristics and payment conditions - Tacit collusion arises from the repeated competition, particularly under FFS payment
Brosig-Koch et al. [13]	CAP, FFS and mixed	- Participants overtreat patients under FFS and undertreat them under CAP, though to a less extent compared to predictions under profit-maximization assumptions - Mixed payment schemes significantly reduce deviation from patient-optimal treatment level and improve patients’ health benefits - Altruistic behavior towards the patients varies on participants background (medical students are found more altruistic than nonmedical students)
Brosig-Koch et al. [14]	Physicians’ preferences for FFS or CAP	- Most participants prefer FFS to CAP, regardless of their previous experience with of the two payment schemes - Subjects preferring FFS are ex-ante and ex-post less patient-oriented than those choosing CAP
Brosig-Koch et al. [15]	Bonus payments and information provision	- Introducing a bonus payment increases the provision of low and high-quality information - When the bonus payment compensates for the cost for providing high-quality information, primary care physicians (PCP) transfer more high-quality information - Information provision does not vary on whether the bonus payment is cost-neutral or not - Designing a bonus payment which exceeds the cost of passing on low-quality information is efficient only if the payment is introduced cost-neutrally - PCP show a more selfish behaviour and provide less-quality information when the specialist, instead of the patient, benefits from information transfer and the specialist earns more than the PCP
Brosig-Koch et al. [16]	FFS and quality competition	- In the absence of competition, FFS drives physicians to choose a higher quantity of medical services than the patient-optimal level - Without competition, average patient-benefit is significantly lower for low-severity patients than for high-severity patients - In the absence of competition, subjects also care about patients’ benefit rather than about their own profit only - Regardless of the severity of illness, active patients are better off when competition is at play, though patients’ benefit is still lower than the optimal quantity - Passive patients are worst off under competition, especially low-severity ones - Competition seems to drive tacit coordination between clinicians
Cox et al. [20]	Hospital readmissions rates and P4P	- While the use of deferred profit-sharing bonus (BO) payments increases readmission rates, the employment of instantaneous profit-sharing bundled (BU) payments does not - The probability of overall unplanned readmissions increases when a patient is discharged earlier than recommended - Pay-for-performance mechanism decreases hospital length of stay, though this effect is more marked when physicians are provided with evidence-based discharge criteria (clinical support system)
Di Guida et al. [27]	FFS with different fee sizes, patient types and market conditions	- Patients are generally overtreated under FFS, though the effect varies on the patient’s type - Decreasing the fee size reduces overprovision, regardless of the patient’s type - Harmed patients, undergoing a decrease in the benefit of care due to overprovision, are at less risk of excess treatment - Resource constraint mitigates overprovision compared to resource abundance
Finocchiaro Castro et al. [30]	Medical malpractice liability and payment schemes (CAP, FFS, and mixed)	- Participants overtreat patients under FFS and undertreat them under CAP - The introduction of malpractice liability increases the amount of services provided regardless of the payment structure - Subjects with a medical background react more aggressively to liability, especially under CAP - The provision of medical services is also affected by the patient’s severity of illness
Godager et al. [39]	Performance disclosure and FFS	- When performance information on physicians’ performance is disclosed to peers, participants’ likelihood of providing services in accordance with the medical norm or with the maximization of the joint benefit (patient and provider's) significantly increases
Green [40]	Financial incentives and P4P, CAP and FFS	- Under retrospective payments systems (i.e., FFS and FFS with P4P) physicians provide more medical services than under prospective payment systems (salary, CAP, CAP with P4P and CAP with report card) - Focusing on the quality of services, retrospective payment systems rule out participants’ intrinsic motivations for patient’s wellbeing
Hennig-Schmidt et al. [46]	FFS and CAP	- Participants overtreat patients under FFS and undertreat them under CAP - Overprovision and underprovision behavior depend on patient’s degree of illness. Under FFS, physicians overserve patients in a good and intermediate health status, while more severe patients and intermediate ones are underserved under CAP - Patients’ benefit loss is larger for patients in a good health status under FFS compared to CAP, while the opposite is true for intermediate and severely ill patients
Hennig-Schmidt and Wiesen [47]	Patient-regarding behavior and FFS and CAP	- Medical students are more patient-oriented in their provision behavior, eventually sacrificing their own profit in favor of patients’ welfare - Nonmedical students provide a higher (lower) volume of medical services under FFS (CAP) compared to medical students - Medical students show to be influenced by the patients’ health benefit in their decisions, while nonmedical students only look at their own payoffs
Hennig-Schmidt et al. [48]	Fraudulent behavior in healthcare and DRG reimbursement rates	- In the absence of audit mechanisms dishonest behavior (obstetrics’ misreporting of child’s birth weight which increases the obstetrics’ payoff) is observed in three-quarters of decisions - Introducing a random audit probability together with a fine reduces upcoding behavior when dishonest report is detectable
Irvine et al. [51]	Prescription to time-inconsistent patients (individual incentive and salary)	- Most participants adapt to patient non-adherence, recommending the medical treatment which maximizes the difference between the short-term pain and the long-term benefit (welfare maximizing), especially under individual incentive - Under salary, the proportion of participants opting for the socially optimal medical treatment decreases throughout the rounds of the experiment - Medical students are more likely to prescribe the welfare maximizing treatment, from the very first round - Medical students and risk-averse individuals are less likely to recommend medical treatments providing low benefits or high costs
Kairies-Schwarz and Souček [53]	P4P with bonus-malus incentives and DRG	- Most physicians choose the patient’s optimal option under DRG, regardless of medical specialty and patient’s severity - The introduction of performance incentives significantly increases the proportion of physicians choosing patient’s optimal treatment only in the presence of high monetary DRG incentives - Hospital physicians are more willing to select patient’s optimal option than medical students - P4P generally yields to the increase in patient-optimal decisions among medical students
Keser et al. [54]	Custom-made healthcare and FFS and CAP	- Participants overtreat patients under FFS and undertreat them under CAP. Overprovision is higher for patients in good health status, while underprovision is higher for high-severity patients - Physicians customize their care according to the payment method - Physicians are not affected by an ex-ante payment reduction in CAP - Patients are worse off under CAP than under FFS
Lagarde and Blaauw [59]	Social incentives and FFS, CAP and salary	- Regardless of patient’s benefits, FFS produces the highest quantity of output, and CAP the lowest - Risk-adjusted CAP avoids patients cream-skimming - The highest quality of output is achieved under salary, followed by CAP - Social incentives (benefits to patients linked to the quality of work) improves providers’ performance
Lee et al. [62]	Prescription and CAP and FFS	- In the absence of monetary incentives, there is no difference in the number of treatments provided - Doctors provide more services under incentives similar to FFS than under incentives similar to CAP - The amount of treatment provided is independent from the perceived severity of the patient’s health status
Li et al. [66]	FFS, DRG, mixed FFS and DRG-based	- Physicians provide a higher amount of services under FFS than under DRG - Physicians provide a higher (lower) amount of services under DRG-based mixed (FFS-based mixed) payment schemes than pure DRG (FFS) - Patients’ health benefits improve under mixed financial schemes compared to pure systems - For high-severity patients a larger deviation from the optimal amount of treatment is observed under pure DRG and DRG-based mixed, while the opposite is found under FFS schemes
Martinsson and Persson [68]	Altruism and FFS and CAP	- Physicians’ attitude towards risk and ambiguity affects their provision of medical treatments under CAP - Most physicians are altruistic towards the patients, though the degree of altruism varies on patients’ need for care - Both pure altruism and pure selfishness are more often observed under FFS than under CAP
Oxholm et al. [70]	Market conditions and CAP	- Underprovision of care in capitation-based scheme to severely ill patients exists regardless of resources availability - High-severity patients benefit the most from a fixed salary to providers under resource abundance, while no difference between patient types is detected under resource constraint - Physicians take better care of patients when differentiations to CAP are introduced, regardless of resource availability
Oxholm et al. [71]	Medical service provision and CAP and P4P	- Patients who can potentially reach the health target benefit from pay-for-performance system by receiving additional care, compared to patients without any potentiality who are provided with less care - Physicians redistribute care between patients in P4P under resource constraint
Reif et al. [74]	Financial incentives and FFS and CAP	- Payment systems affect physicians’ reporting and provision behavior - Physicians care about the payoffs of a third party which funds medical service provision and reduce the amount of services provided to save costs for the third agent - Participants are more patient-oriented in the medical framing, compared to the neutral framing
Waibel and Wiesen [85]	Referral rates	- Diagnostic effort does not vary on the referral fees - High referral rates increase referrals regardless of physicians’ degree of altruism - Compared to a baseline condition without referral fees, the introduction of medium-size referral rates incentives referrals for barely altruistic primary care providers of severely ill patients
Wang et al. [86]	Patient-regarding preferences and FFS and CAP	- Patient-regarding motivations do not differ across the subject pools (Chinese and German physicians, Chinese medical students) - Experience makes physicians rational in the decision-making
Wettstein and Boes [88]	Pharmaceutical pricing and salary and bonus	- Most players submit at least one consistent offer (i.e., below the reservation price for buyers and above for sellers) - Buyers’ reservation price is higher in the real payoff price group than in the fictive real- world price one - Price offers and margins between price groups increase throughout the game - Sellers’ price offers are significantly higher in 4/5 rounds in the fictive real-world price framing than those of the buyers - The real payoff price group is significantly more successful in concluding negotiations than the fictive real-world price one - Considering the possible trades, the proportion of successful negotiations increases throughout the game, regardless of the price group, maybe due to the increase in the bonus provided for successful trades
Wettstein and Boes [90]	Pharmaceutical pricing and salary	- In the absence of any alternative, buyers and sellers are able to conclude the negotiation, regardless of the treatment - In the cost–benefit treatment, no agreement is reached when an alternative option is available - The value for money of an alternative positively impacts on the offers - The patient’s final benefit or the additional cost for the negotiated option negatively impacts on the offers - The expected reimbursement price, the patient’s final benefit and the value for money are higher on average in the control group than in the risk-taking group
Zhang et al. [91]	Altruism and FFS, DRG and mixed	- Deviation from the provision of the patient-optimal level of treatment is larger under DRG than under FFS, especially for male students - Introducing mixed payment schemes reduces deviation from patient-optimal level of care (underprovision in DRG and overprovision in FFS), with respect to pure payment systems - Reduction of suboptimal behaviours in DRG-based (FFS-based) mixed system is higher the lower the DRG (FFS) component - Patient’s severity of illness increases underprovision (overprovision) in DRG (FFS) payment schemes - Suboptimal behaviours in the provision of services are less likely among students with internship experience, under DRG systems - Altruism decreases when moving from pure systems to mixed schemes - Medical students’ altruistic parameter varies on the trade-off range between their own profit and patient’s benefit - More altruistic students generate higher patients’ benefit, especially under pure payment systems
Zhang et al. [92]	Altruism and FFS	- Highly altruistic medical students show a marked preference for non-financial attributes (opportunities for career development, work environment, training) in their job choices - Less altruistic students are more likely to opt for high-income specializations

Most of those experiments have been run with students, whereas few of them have involved medical students (see for instance [20, 59, 71]) and physicians (see for instance [74, 86]).

Although not all papers assess payment schemes as the main objective of their research question, a relevant portion of works focuses on physicians’ behavior in medical service provision under different payment schemes. Hennig-Schmidt et al. [46] have been the first to test the theoretical predictions introduced by Ellis and Mcguire [29]’s seminal model showing that physicians’ treatment decisions are affected by payment systems. Thus, in Hennig-Schmidt et al. [46], participants, acting as physicians, choose the amount of services to provide to standard patients, varying on the severity of illness (i.e., low, medium, and high), under alternative payment schemes, capitation (CAP) and fee for service (FFS), respectively.^13^ Brosig-Koch et al. [11] compare medical and nonmedical students’ behavior, showing that the former are less affected by financial incentives than the latter.^14^ The experimental design of Hennig-Schmidt et al. [46] has been replicated by several other authors to test the impact of different monetary incentives, such as mixed systems [12–14], report cards [40], salary [59] and diagnosis-related-group (DRG) [66]. Similarly, 12–14 test whether subjects’ *ex ante* preferences for either CAP or FFS, elicited through the strategy method [81], can justify their *ex post* treatment decisions in the lab. Using the same payment schemes, Hennig-Schmidt and Wiesen [47] measure patient-regarding motivations among medical and nonmedical students, showing that medical students are more altruistic and prone to sacrifice their own profit compared to nonmedical ones. Still focusing on CAP and FFS, Keser et al. [54] make participants progressively face a reduction in the lump sum payment (i.e., CAP) to test whether physicians react by customizing care at the individual patient level and whether this result is also observable under FFS. Inspired by 12–14, Zhang et al. [91] demonstrate how the shift from pure payment systems (either FFS or DRG) to mixed ones, with DRG and FFS components in different weights, increases patients’ benefits (confirming [66]. The average value of the altruistic parameter found by Zhang et al. [91] approaches that found in 12–14 , but it is still lower than that obtained in Zhang et al. [92]. Using a combination of surveys, discrete-choice experiments and an online experiment,^15^ Zhang et al. [92] demonstrate that prospective physicians with low altruistic motives would opt for high-income specialties and would be less prone to accept job in rural areas, confirming a wide range of literature [6, 59, 63]. Patient-regarding preferences are, instead, analyzed in Wang et al. [86] comparing medical students and real physicians, to show that such preferences are not significantly different across the subject pools, differently from what found by Hennig-Schmidt and Wiesen [47] and Kairies-Schwarz and Souček [53].

Additionally, also drawing from Hennig-Schmidt et al. [46]’s model, Finocchiaro Castro et al. [30] introduce a random probability for a physician to be sued for malpractice to test its effect on medical service provision, whereas Martinsson and Persson [68] propose a patient health benefit function to show how physicians’ altruism varies on patients’ medical needs. Under the same design, Godager et al. [39] prove that disclosing information on providers’ performances to their peers benefits the quality of care under FFS.

Departing from Hennig-Schmidt [46], Di Guida et al. [27] investigate how physicians under FFS allocate services to patients with different responsiveness to treatments throughout 36 working days, highlighting that resource constraints might be a deterrent to overprovision. The influence of resource limitations on physicians’ patient prioritization is confirmed by Oxholm et al. [70] in their laboratory experiment, where medical students have been incentivized by CAP, differentiated CAP (i.e., the fixed amount vary with the patients’ needs), and salary. Similarly, Oxholm et al. [71], distinguishing patients by treatment responsiveness, demonstrate how redistribution of services is stricter under pay-for-performance when resource constraints are at play.^16^ Resorting to the same payment system with bonus-malus incentives in contrast to a simple DRG system, Kairies-Schwarz and Souček [53] find that the former improves the quality of care depending on the fee size of DRG as well as on physicians’ initial orientation towards the patient. Coming back to CAP and FFS, Lee et al. [62] show how doctors’ number of prescriptions are affected by monetary incentives rather than by patients’ severity of illness.

Differently from previous works, Reif et al. [74] account for the presence of an insurer who must budget for physicians’ cost of providing services to patients whose status of health can be misreported. Dishonesty is also investigated by Hennig-Schmidt et al. [48] who underline the need to introduce audit probability to avoid fraudulent behavior in reporting information (i.e., obstetricians reporting birth weights), which determine reimbursement rates.^17^ Referral rates are instead the focus of Waibel and Wiesen [85] who show that when referral fees are increased, the number of referrals raises regardless of the patient type. The introduction of bonus payment for information provision while referring the patient to the specialist is the purpose of Brosig-Koch et al. [15]. Subjects playing the role of primary care physicians (PCP) decide whether or not to pass low/high-quality information, while the specialist automatically provides the optimal treatment to the patient. Different experimental conditions are tested: change in the beneficiary of information (specialist vs patient), change in the relative payoff of the PCP and the specialist, change in the bonus and in the additional capitation payment. Supporting the authors’ theoretical model, data show that introducing the bonus payment incentive increases PCPs’ likelihood to provide information. Differently, payment systems variations are used to assess hospital readmission rates by Cox et al. [20], demonstrating that pay-for-performance incentives together with decision support system drive to more cost-effective discharge decisions. The effectiveness of pay-for-performance is also confirmed in the laboratory experiment conducted by Bardey et al. [7], assessing the impact of monetary incentives on the use of personalized medicine. Irvine et al. [51] discuss how participants react to computer-based patients’ non-adherence to medical prescriptions, under two different payment conditions. Under the first payment condition (named the individual incentive) physicians are paid whether the patient conforms to the treatment recommendation, under the second one, physician receives a salary which is independent from patient’s outcome. Differently from the above-mentioned topics, two pharmaceutical pricing options are investigated in the online experiment by Wettstein and Boes [90], a cost–benefit measure and an outcome based one, respectively. Participants must buy or sell^18^ a closed envelope containing a donation to the patient association of an unknown amount, or eventually opt for an alternative with known price and patient’s benefit. Subjects are given a salary for completing the task. Depending on the treatment, in addition to reaching an agreement on the price, participants are required to either agree on the resulting patient’s benefit (otherwise the price of the concluded negotiation is cut in half) (cost-sharing treatment), or to estimate the donation contained in the sealed envelope (risk-sharing treatment). The outcome of the negotiations both in terms of patient’s benefit and offer prices are significantly affected by the existence of available alternatives. Wettstein and Boes [90] draw their experimental design from Wettstein and Boes [88] who, rather than evaluating the impact of value-based interventions, focus on just the impact of negotiation for life-extending drugs on societal outcome. Here, in addition to salary [90], participants may also receive a bonus which depends on their preferences, the offer and the counteroffer prices. Furthermore, participants are divided into two groups according to different magnitude price framings: the 100 k$ group with fictive real-world prices and the 1$ group with real payoff prices. Results, which are then confirmed in the follow-up study [89],^19^ show that offer prices and successful negotiations depend on the price magnitude framing.

Finally, moving to another common topic, financial incentives are used as a tool to investigate how competition between providers affects physicians’ provision behavior under CAP and FFS [12–14]. Similarly, Brosig-Koch et al. [16] test how physicians, incentivized by FFS, respond to competition, facing an heterogenous patient population. Here, patients differ on health status (high/low severity), which represents the novelty with respect to Brosig-Koch et al. [12–14], and responsiveness to the quality of services provided.^20^ Data show that patients’ reactivity to treatment crucially determines the effect of competition among clinicians.

Table 3 indicates whether for each of the study a theoretical model is reported and whether its predictions are fully or partially confirmed by the experimental results. What clearly emerges from the use of experimental methods in the context of financial incentives in health is that providers are not uniquely driven by monetary rewards. The theoretical model provided by Ellis and Mcguire [29] suggests that remunerating physicians through prospective payments system (e.g., FFS) would lead to the overprovision of health services, while the reverse would take place under cost-shared schemes (e.g., CAP). Although the above-mentioned predictions are confirmed in the experimental context, they attenuate in the presence of additional factors which cannot be accounted for in a formal paradigm. First, it must be noticed that incentive schemes are not uniformly perceived by physicians, but depend on their working experience and on their degree of altruism [47, 86]. Additionally, reactions to incentives are not so clear-cut when providers have the possibility of referring the patients to specialists or when they are informed about their peers’ performances or when they face budget and resource constraints [39, 70, 85]. Furthermore, providers take their decisions under given economic incentives also taking into account patient’s severity of illness and his reactivity to treatment [16, 27, 71]. The above-mentioned aspects are difficult to include in a single theoretical model. This explains how the resort to experimental methods allows not only to test pre-existing theoretical predictions but also to derive insights to inform policy decisions.

**Table 3:** Consistency between theoretical predictions and experimental results for studies on payment schemes; *Source*: our elaboration

Authors	Formalized theoretical model	Consistency between theoretical predictions and experimental results
Bardey et al. [7]	Yes	Yes
Brosig-Koch et al. [11]	No	–
Brosig-Koch et al. [12]	Yes	Partially
Brosig-Koch et al. [13]	Yes	Yes
Brosig-Koch et al. [14]	No	–
Brosig-Koch et al. [15]	Yes	Partially
Brosig-Koch et al. [16]	Yes	Partially
Cox et al. [20]	No	–
Di Guida et al. [27]	No	–
Finocchiaro Castro et al. [30]	Yes	Yes
Godager et al. [39]	No	–
Green [40]	No	–
Hennig-Schmidt et al. [46]	Yes	Yes
Hennig-Schmidt and Wiesen [47]	No	–
Hennig-Schmidt et al. [48]	No	–
Irvine et al. [51]	Yes	Yes
Kairies-Schwarz and Souček [53]	No	–
Keser et al. [54]	Yes	Partially
Lagarde and Blaauw [59]	Yes	Partially
Lee et al. [62]	No	–
Li et al. [66]	No	–
Martinsson and Persson [68]	No	–
Oxholm et al. [70]	No	–
Oxholm et al. [71]	Yes	Partially
Reif et al. [74]	Yes	Partially
Waibel and Wiesen [85]	Yes	–
Wang et al. [86]	Yes	Partially
Wettstein and Boes [88]	Yes	Partially
Wettstein and Boes [90]	Yes	Partially
Zhang et al. [91]	No	–
Zhang et al. [92]	No	–

### Other topics in the provision of health services

There are several papers in our pool which cannot be inserted into a specific group, facing a variety of health topics such as resource allocation, health insurance decisions, competition and so on. Table 4 summarizes the specific topic investigated and the main results of each paper.

**Table 4:** Other topics in the provision of health services explored in the sample; *Source*: our elaboration

Authors	Topic	Results
Ahlert et al. [1]	Distributive behavior	- Economists behave more often as payoff maximizers in the neutral framing than in the medical one - Physicians are less sensitive to the experimental setting but are more willing to maximize their payoffs in the medical frame. However, they are generally more likely to maximize the number of recipients or to behave according to the Rawlsian rule
Ahlert et al. [2]	Distributive behavior	- Most participants behave according to either the ‘truncated split’ (TS) (first allocating the minimal amount to all the recipients and then the remaining part equally) or the ‘truncated utilitarian rule’ (TU) (allocating in order to maximize the sum of payoffs of all receivers, after having allocated the minimal amount) - Economics students are more focused on payoffs and their maximization (TU), while law and medical students are more concerned about the equalization of resources to be distributed after giving all the minimal amount (TS) - When resources are not enough to satisfy all recipients, students with medical background opt for equally splitting resources, avoiding any waste (leximin rule)
Angerer et al. [4]	Monitoring	- In the absence of liability and verifiability, undertreatment and overcharging are detected - Both endogenous monitoring and exogenous monitoring reduce the level of undertreatment and overcharging observed and improve market efficiency
Arrieta et al. [5]	Risk preferences	- Risk tendencies are health-context specific - Students with a medical background are more risk-averse, especially in the health domain - When subjects decide for others, introducing benefits to the third party reduces the level of risk aversion
Attema et al. [6]	Patient-regarding altruism	- Medical students tend to decide in a patient-regarding way - Medical students attending the pre-clinical phase are more profit focused than their peers in the first week of their studies, but less profit-oriented than their colleagues in the clinical phase. Patient-regarding altruism slightly returns to increase in the practical study phase - Male students are more profit-oriented than female ones - Medical students’ preference for altruism is linked to their specialty decisions - Nonmedical students are less altruistic than medical ones
Brendel et al. [9]	Resource scarcity and prioritization decisions	- Most participants allocate a constant portion of their budget to patients and then reduce the amount of services provided in response to significant budget reductions - Most physicians provide equal benefits between patients - The less resources available the less patient benefits
Brock et al. [10]	Generosity and pro-social behavior	- Physicians who show pro-social behavior in the laboratory are also generous in their normal practices - Physicians provide better performances in response to peers’ monitoring and encouragement. This effect is equally shared between generous and ungenerous clinicians
Cao and Liu [17]	Multitasking and diagnostic decision making	- Strategy of diagnostic decision is not significantly affected by concurrent tasks - Diagnostic decision time increases in the presence of concurrent tasks - The presence of a concurrent memorization task significantly reduces diagnostic performance, both in terms of reaction time (this is also true for the concurrent monitoring task) and accuracy in responses - Performing a concurrent task significantly increases mental workload, regardless of the task complexity
Cox et al. [21]	Hospital discharge	- Clinical decision support systems (CDSS) in the form of recommendations on patients’ discharges reduce readmissions rates and patients’ length of stay - CDSS promote time efficiency in making discharge decisions and improve participants’ performance as measured in terms of experimental earnings - Subjects perform better under time constraint - Subjects appreciating CDSS are more likely to provide better discharge decisions
Ge and Godager [35]	Strategical decision-making and market competition	- Patients benefit from larger competition - Higher competition drives to individuals’ deterministic behavior
Ge et al. [36]	Cost-sharing	- Medical students are concerned for patient’s health benefit and consumption opportunities after cost-sharing, when deciding on treatment options - When the profit for the physician is high or the patient’s benefit from the treatment (either in terms of health benefit or consumption opportunity) is low, medical students are more willing to give up their profit to increase patient’s utility
Greiner et al. [42]	Prescription behavior	- Separating prescription and treatment activities between physicians reduces overtreatment behavior and increases patient’s willingness to accept severe treatments - When physicians provide diagnosis free of charge, undertreatment is observed, together with patients’ reluctance to accept mild treatments
Han et al. [43]	Quality competition and hospital mergers	- Average quality following a merger is lower than pre-merger quality - Participants’ choices on quality level are significantly higher than predicted for pure profit-maximizer hospitals (maybe due to altruistic behavior towards the patients) - When the merger leads cost synergies, average quality choices increase compared to the scenario without synergies - Results do not change between individual and team decisions
Herr and Normann [49]	Organ donation	- Two-thirds of the participants show stronger preferences for the priority rule (a reciprocal rule which prioritizes registered donors) - Priority rule increases donors’ registrations - Medical students register more often as donors and opt for the priority rule more frequently compared to students with other backgrounds - When asked about the desirability of the priority rule in the field, participants of the donation experiment vote for such rule more often than non-participants
Huck et al. [50]	Medical insurance	- Under the insurance condition (patient shares the cost of the treatment with all the other patients), patients more frequently consult the physician, while physicians are more likely to overtreat the patients - The introduction of competition (patient can choose which physicians to be assigned to) mitigates excess consultations and overtreatment - When insurance and competition interact, efficiency increases and patients are likely to receive the required treatment
Kessler and Roth [55]	Organ donation	- When information about the others’ donations and use of the loophole rule (i.e., subjects can register to get a priority but simultaneously refuse donating organs) are made public, subjects are less willing to donate - The priority allocation rule increases donations
Kesternich et al. [56]	Professional norms and physicians’ behaviour	- There is a strong effect of the Hippocratic Oath on the provision of good, since it increases participants’ altruism towards the receiver, especially in the medical framing - Participants are more willing to provide good when the receiver is a real hospice, regardless of treatment conditions - When the third-party payer is introduced, the more of the good is provided by participants, the more they themselves earn from the provision - The salience of the Hippocratic Oath makes subjects more prone to provide good regardless of efficiency concerns
Kolstad and Lindkvist [58]	Prosocial behavior and self-selection in the public sector	- Medical students preferring to work in the public sector show more pronounced pro-social preferences than those opting for the private sector
Laker et al. [60]	Information overload and clinical decisions	- Decision quality increases when emphasis framing (underlining of some parts of the information on the patient’s condition) is introduced - Emphasis frame increases the percentage of physicians correctly diagnosing the hypothetical patient - Decision time significantly increases with emphasis frame
Li et al. [63]	Altruistic and equality-efficiency preferences	- Medical students are less altruistic and more efficiency-oriented than the average American population - Medical students opting for low-income specialties are more altruistic than their peers choosing high-income specialties - Students from top-ranked faculties exhibit similar social preferences to a sample of elite law students - Participants’ socio-demographic characteristics (age, race, marriage status, parental education) help to predict altruistic behaviour [64]
Li et al. [65]	Altruistic and equality-efficiency preferences	- Physicians are more altruistic than any other population (e.g., a representative sample of US adults, an elite sample of wealthier and educated individuals, a sample of medical students) - Physicians’ equality-efficiency orientation is not significantly different from that of the general population - Medical students are much more efficiency-oriented than practicing physicians
Martin-Lapoirie [67]	Teamwork and Medical Malpractice	- Strict liability and the negligence rule (i.e. the physician has to compensate the patient only if he has demonstrated negligence in at least one consultation) lead to similar precaution behaviors - Healthcare providers choose a positive precaution behavior even in the absence of liability - Strict liability is less efficient than the negligence rule in reducing deviations from social optimal precaution level - Teamwork reduces precaution behavior under liability rules
Mimra et al. [69]	Second consultations	- Introducing the possibility for a patient of asking for a second opinion at a cost reduces overtreatment - Reduced search costs incentivize second consultations and improves market efficiency. The reduction in the cost of treatment outweighs the increase in search costs
Raptis et al. [73]	Ambiguity aversion	- In the health domain a larger number of physicians opts for the treatment with unknown probability of survival, compared to the financial domain - Younger physicians (< 50) exhibit higher ambiguity aversion than their older colleagues - Ambiguity aversion in the financial domain is associated with correctly recommending therapy
Saposnik et al. [80]	Ambiguity aversion	- Ambiguity aversion is higher in the financial domain than in the health domain - High ambiguity aversion in the financial domain is associated with therapeutic inertia

For instance, Ahlert et al. [1] ask economics and medicine students to allocate a given amount to seven potential recipients varying in the quantity needed to obtain a positive payoff, either in a neutral or in a medical^21^ framework. Results show that economists are significantly affected by the experimental setting, mimicking more often payoff-maximizers’ behaviors in the neutral framing than in the medical one. Economics, law and medical students face similar tasks in the questionnaire experiment by Ahlert et al. [2].^22^ Here, a significant difference between medical and economics students in allocation decisions is clearly observed, with law students making choices close to those of their medical colleagues. Brendel et al. [9] check how resource scarcity impacts on medical service provision. Medical and nonmedical students in the role of physicians decide how many services to provide to patients with varying characteristics, under different budget constraints. Results reveal that patients’ health benefits decrease in response to more severe budget limitations, receiving fewer services.

To address the role of altruistic preferences^23^ in medical decisions, Kolstad and Lindkvist [58] combine the results of a dictator game and medical students and nurses’ responses to a questionnaire to investigate whether their social preferences affect their willingness to work in the public or private sector in Tanzania. Results demonstrate that medical students preferring to work in the public sector show more pronounced pro-social preferences than those opting for the private sector (see [92]). In the same setting, Brock et al. [10], merging a laboratory experiment and data from the field,^24^ measure clinicians’ generosity through a dictator game where the clinician takes the role of the dictator and the participant from the standard subjects’ pool stands for the receiver. Data show that the majority of physicians equally divide the allocation between themselves and the other person. Similarly, Kesternich et al. [56] investigate how medical students trade their own profit, the patient’s benefit, and the third party’s payment for medical treatment, in the context of professional norms. After being endowed with a different version of the Hippocratic Oath, participants play eight standard dictator games and four cost dispersion games.^25^ Treatments vary on the salience of professional norm, the framing (neutral vs medical), and the identity of the receiver (a student vs a real charity). The introduction of the Hippocratic Oath is found to increase participants’ altruistic motivations. A graphical version^26^ of the dictator game is used in the web-based experiment by Li et al. [65] to investigate physicians’ altruism and equality-efficiency orientation.^27^ US practitioners from different specialties are asked to distribute real money between themselves and an anonymous party. Additionally, participants face the cost of giving to the other side, which varies across the allocation decisions. Results are compared with data from previous experiments and show that physicians are more altruistic than both the sample population and a cohort of medical students, but less efficiency-focused than medical students. The above-described methodology was already adopted in Li et al. [63] to study the social preferences of first to fourth-year medical students from US (see also [64]^28^). Data show that medical students are significantly less altruistic and more efficiency-oriented than the average American population. Moreover, by comparing students from top-ranked universities with students from low-ranked universities, the former are less altruistic than the latter and exhibit social preferences like a pool of elite law students. Differently, in Attema et al. [6], combining lab and online experimental sessions, German medical students with different seniority decide between two treatment alternatives for 30 stylized patients, where the two choice options represent the trade-off between patient’s benefit and physician’s profit.^29^ Generally, students tend to be patient-oriented in their decisions, although their altruism declines throughout the seniority. Patient-regarding behavior significantly differs between medical and nonmedical students, with the former being more altruistic than the latter. Finally, prospective physicians with higher-income expectations put less weight on patient’s benefits, with respect to their own profit (confirming [92]).

Physicians’ patient-regarding preferences with a specific focus on cost-sharing are discussed in Ge et al. [36], combining methods from discrete-choice (i.e., to design choice menus) and health economics experiments. Medical students make 23 treatment choices based on two alternatives for a hypothetical patient who has to pay the out-of-pocket fee ‘required’ for the treatment received. The two options differ in terms of physician’s profit, patient’ health benefit and patient’s consumption opportunities after cost-sharing. In this way, participants’ decisions determine the co-payment and the money available to the patient after treatment, which is the difference between the initial endowment and the co-payment. Data demonstrate that medical students care about both patients’ health benefit and consumption opportunities, although the former prevails on the latter in driving treatment decisions.

Moving to health insurance, Huck et al. [50] investigates the effects of both insurance and competition on the interaction between patients and physicians. Patients, who pay the whole cost of the treatment or share the cost with all the other patients in the insurance condition, can choose whether to consult a physician and eventually which physician to refer to in the competition condition. The physicians, instead, choose the treatment to provide. Under the insurance condition, patients consult the physician more frequently, whereas physicians are more likely to overtreat the patients. The last result is mitigated when competition is introduced. The effects of market competition on medical treatments are assessed in Ge and Godager [35]. Participants acting as physicians select the medical services to provide under three different market conditions: monopoly, duopoly and quadropoly. Results show that participants are more patient-oriented in their decisions when competition is higher. The outcomes of a hypothetical merger among competing hospitals are discussed in Han et al. [43]. Participants in the role of a hospital head decide on the quality of services to provide to patients before and after eventually experiencing a merger. Participants’ selections reveal that quality does not benefit from merging. Close to competition issue, Mimra et al. [69] address the role of second consultations in a lab experiment where participants are randomly assigned the role of physicians or patients. The former decides whether to overtreat a patient, the latter can eventually ask for a second consultation at a high or low cost depending on the treatment. Overtreatment is mitigated under the second consultation condition. When search costs are reduced, patients overuse second opinions.

Martin‑Lapoirie [67] check how teamwork among healthcare providers affects the individual precaution behavior under different liability scenarios. Subjects playing as healthcare professionals select the effort level for each consultation, while dummy patients decide whether to refer twice to the same physician or to consult two different physicians. Results show that strict liability and the negligence rule^30^ lead to similar precaution behaviors. In their laboratory experiment, Angerer et al. [4] investigate how introducing the possibility for physician of being monitored either randomly or upon the patient’s request can avoid misbehaviors such as undertreatment, overtreatment, and overcharging. Data show that both endogenous monitoring and exogenous monitoring succeed in reducing the level of undertreatment and overcharging observed and improve market efficiency. To improve decisions, Cox et al. [21] investigate how introducing clinical decision support system (CDSS) affects physicians and fourth-year medical students’ hospital discharge decisions. Recommendations provided by CDSS contain patients’ probability of readmission in case of an incorrect early discharge decision, which is costly to the provider. Results provide evidence for CDSS as an effective tool to improve discharge decisions.

Prescription behavior is the focus of Greiner et al. [42], who test the possibility of separating prescription and treatment activities through a lab experiment. In the baseline condition, the physician decides the prices for possible treatments, while the patient decides whether to consult the doctor and whether to undertake the suggested treatment*.* Under a different experimental condition, the patient interacts with two different doctors: the first one is only in charge of the prescription phase (for free), the second one only implements the treatment previously prescribed. Although this second condition results in a reduction of overtreatment, it reduces efficiency due to miscoordination between the doctors involved.

Cao and Liu [17] study how concurrent tasks impact on diagnostic decisions. Participants play three single task conditions and two dual task conditions. Task conditions include: a visual task (abstract diagnostic decision-making task), and two auditory tasks (a sound monitoring and a memorization task). In each task, participants, after eventually asking for additional diagnostic tests, are asked to indicate the disease which the hypothetical patient suffers from. Diagnostic performance is worsened in the presence of simultaneous tasks. The effect of information overload on clinical decision-making is addressed in Laker et al. [60]. Real physicians after looking at a fictitious medical scenario are asked to report the preferred care plan to the hypothetical patient. In the experimental condition, physicians can benefit from emphasis frame, which is the marking of salient components of the information provided on patient’s medical scenario, to minimize the effect of information overload.

Organ donation is addressed in Kessler and Roth [55] and Herr and Normann [49]. In the former, college students play a game where they have to opportunity to register as organ donors, although instructions are neutrally framed, under different allocation rules. Results demonstrate that the presence of a loophole, where subjects can register to get a priority but simultaneously refuse donating organs, has a detrimental effect on the donation resulted by the priority rule. In the latter experiment, medical and nonmedical students first join several rounds of a donation game and, after having already tested it, they are asked to vote for the implementation of a priority rule in the last rounds of the game. Two-thirds of the participants show stronger preferences for the priority rule.

Finally, the last papers included focus on participants’ risk and time preferences measurement. As reported in the literature, risk preferences are domain-dependent (see e.g., [87], Weber et al. 2002), and then, several authors prefer measuring risk across different contexts before drawing conclusions. For instance, in their laboratory experiment, Arrieta et al. [5] measure medical and nonmedical students’ risk preferences in deciding for others both in the monetary and health domain, using the Holt and Laury (2002) (HL)’s multiple price list method. Participants playing the role of a physician who takes decisions in three different health contexts, must choose the treatments to provide to patients. Depending on the context, health gains can be expressed in terms of years of life for a patient with varying health conditions or hours of pain alleviated. Results confirm that risk tendencies are health-context specific. Additionally, students with a medical background are found to be more risk-averse than their peers and surprisingly such attitude is exacerbated in the health domain. Similarly, Rapis et al. (2017) combine simulated vignettes, surveys, and behavioral experiments to study the association between clinicians’ risk preferences and therapeutic prescriptions in atrial fibrillation. In the experiment, physicians are asked to select either a visual option with known probabilities of the outcomes or an alternative option with unknown probability of the same outcomes, with a gray bar indicating the degree of uncertainty of the winning probability in the second option. Second, physicians are asked to make a similar choice in the health context, with a gray bar indicating the degree of uncertainty of the survival probability. Data show that physicians are more willing to select ambiguous options in the health domain than in the financial domain. The above-mentioned results differ from the ones of Saposnik et al. [80].^31^ Using the same combination of methods of Rapis et al. (2017) and adding risk aversion measurement, Saposnik et al. [80] find that neurologists are more reluctant to choose ambiguity options in the health domain. Finally, high aversion to uncertainty leads to treatment inertia in the management of multiple sclerosis.^32^

## Concluding remarks

Our study provides a systematic review of the literature applying behavioral and experimental methods to health issues related to different perspectives in the provision of health services. This has not been an easy task. Many studies have been incorrectly classified as ‘*experiments in behavioral health*’, although their designs are not incentive-compatible and do not provide real consequences for participants [37]. Thus, of the 1248 articles retrieved, published between January 2011 and July 2023, only 56 articles have met our inclusion criteria. Specifically, we have focused only on laboratory and online (hypothetical lab) experiments, excluding field experiments, discrete-choice experiments, control random trials, and quasi-experiments based on hypothetical choices or stated preferences due to the absence of any monetary incentive.

The selected papers have been first classified according to the object of analysis. A large portion of the 56 papers investigate the issue of payment schemes, whereas the remaining studies focus on several different themes such as health insurance, organ donation and market competition, making it impossible to group them into specific categories. Then, for each paper, we have checked the number of participants and their type (student, medical students, or physicians) describing the experimental designs and main results.

The main aspect emerging from our systematic review on the provision of health services in the lab is the need to involve more physicians in health-related experiments, in order to increase the external validity of the results. Although we are fully aware of the difficulty in gathering physicians in a lab due to their high opportunity cost, their awareness of medical procedure and their experience can make experimental results much more sound and able to provide robust health policy implications. Online experiments can be useful in mitigating such issue, allowing to involve larger sample of physicians reducing their opportunity cost but at the expense of a partial loss of the experimental control. We acknowledge that some researchers argue that physicians’ participation typically concerns field experiments more than laboratory ones. However, some experimental papers show that choosing medical students, or even nonmedical students, to act as physicians in health-related decisions concerning patient treatment may affect the external validity of the results [1, 2, 5, 6, 47]. In this regard, both Brosig-Koch et al. [11] and Finocchiaro Castro et al. [31] show that subjects’ answers to incentivized choices vary on their background and that physicians more easily grasp the main incentives in the experimental designs. The authors conclude that experimenters need to carefully select their pools before testing any health economics prediction.

Another aspect raised by our systematic review is the poor connection between two fields of research: behavioral and experimental economics on the one side and health economics on the other side [37]. Such gap is confirmed by the lack of incentive compatibility typical of many discrete-choice and quasi-experiments. As suggested by Gibson [37], some of the experiments carried out in specific areas (i.e., decisions about health-related behaviors such as smoking, diet, and alcohol drinking) can be improved with the introduction of behavioral consequences for the participants’ stated preferences, providing the appropriate incentive compatible scheme for each area to be investigated. In fact, the variety of health-related topics so far addressed provides evidence of the many advantages of the use of experimental methodology. First, laboratory experiments are replicable, which means that multiple sessions can be run in different times and contexts, also allowing the recruitment of different subjects’ categories (e.g., in terms of age, gender, work experience, specialties and so on). Second, using dummy players (i.e., computerized) or assigning real participants to different roles, permits to simulate real-world interactions (e.g., patient vs doctor, PCP vs specialist), which are generally harder to observe in the field. Finally, multiple stages experiments allow to address several topics at once (e.g., altruism and competition), exploiting responses from the same subjects.

As discussed by Hansen et al. [44], when studying the decision-making of doctors or medical students, a combination of non-experimental methods (e.g., surveys, questionnaires) and economic experiments should be preferred. In fact, surveys and focus groups may represent informative preliminary steps for the experimenters when they need to know more about how doctors make decisions and their decision-making environment before building the experimental designs. Merging different methodologies may overcome the lack of connection between experimental economics and health economics.

Hence, in this systematic review, we have attempted to offer a comprehensive review of a strand of literature dealing with issues related to the provision of healthcare services. This is an area that has significantly grown in the last 10 years, and, to the best of our knowledge, it has not yet been properly reviewed. Although our work shows that the role of incentives related to payment systems is the most investigated strand, there is still much to be done. For example, it is still poorly understood how in P4P systems physician’s behavior is influenced by base payment (FFS or CAP), how patient’s characteristics influence prioritization decisions, and which payment system design features could potentially influence treatment decisions and improve the quality of care for different types of patients.

Additionally, although many areas of research have been explored using laboratory experiments, other areas remain still untreated. For example, to the best of our knowledge, no study has investigated waiting lists from the perspective of healthcare providers yet, though the subject has been widely treated in the health economics literature. Another promising and yet little explored area of research concerns the behavior of providers when there are peaks in demand or under extreme conditions such as pandemic situations.

Finally, some limits of our systematic review are worth mentioning. First, the literature selection process might be limited by the exclusion of some relevant articles which are not contained into SCOPUS database. Additionally, we might have missed other studies due to our keyword selection or to the restricted time span. Furthermore, despite having used all the precautions specific to the systematic review approach to allow for replicability, a certain degree of discretion cannot be neglected. Excluding field experiments as well as experiments on health-related behavior is a critical decision for our systematic review. Consequently, despite transparently explaining the reasons behind our choices, we are conscious that other researchers may have opted for different solutions.

## Footnotes

1 We are conscious that the degree of control available into the lab is partly lacking over the Internet [19].

2 Notice that the current search is the update of a previous version, run in 2022.

3 The baseline implemented search string was: (TITLE (lab* AND experiment* AND physician*) OR TITLE (experiment* AND economic* AND physician*) OR KEY (lab* AND experiment* AND physician*) OR KEY (experiment* AND economic* AND physician*) OR ABS (lab* AND experiment* AND physician*) OR ABS (experiment* AND economic* AND physician*)) AND PUBYEAR > 2010 AND (LIMIT-TO ( LANGUAGE, "English")) AND (LIMIT-TO ( SRCTYPE, "j")).

4 The vast majority of studies were excluded because they did not deal with the experimental methodology: qualitative studies, literature reviews, semi structured interviews, retrospective and cohort studies, cost-effective analyses, stated preference experiments.

5 In a natural experiment, subjects do not know they are in an experiment, so the experimental control is fully lacking [45].

6 We are aware that the exclusion of important research streams such as field experiments as well as experiments on health-related behaviors is a critical decision for our systematic review and that other researchers may have opted for different solutions. However, we believe that our choice allows for a clearer picture of the results of a specific part of the literature that looks at the supply side.

7 Specifically, once the final sample of papers to be included in the systematic review was detected, one of the reviewers had a look at the reference lists contained in each of the selected papers. Whenever a reference which was not included in the Scopus sample was found and was considered to be potentially eligible according to the abstract, its full-text was read simultaneously by the two remaining reviewers for a final decision, based on our inclusion/exclusion criteria. As usual in systematic reviews, a third reviewer was involved in case of disagreement between the first two reviewers.

8 Notice that we have not included the 29 physicians joining the artefactual field experiment in Brosig-Koch et al. [11], for the reasons mentioned in the introduction.

9 For reasons of space and synthesis, additional bibliometric analyses have not been included in the paper but are available from the authors upon request.

10 This is due to our sample restrictions which excluded field experiments but also experiments focusing on nudges on the demand side.

11 https://www.sciencedirect.com/journal/journal-of-economic-behavior-and-organization/vol/131/part/PB.

12 https://onlinelibrary.wiley.com/toc/10991050/2017/26/S3.

13 Notice that data from Hennig-Schmidt et al. [46] are also used by Godager and Wiesen [38] to test physicians’ altruism.

14 Merging data from the artefactual field, physicians are found less sensitive to payment incentives than students and medical students. The size of the effect of the subjects’ pool mirrors Hennig-Schmidt and Wiesen [47].

15 See Rapis et al. (2017) and Saposnik et al. [80] who also combine different methods, in the next section.

16 See Brendel et al. [9] on the same topic in the next section.

17 See Angerer et al. [4] on a similar topic in the next section.

18 Buyer and seller mimic a health minister regulator and a representative of a pharmaceutical company, respectively [88].

19 Wettstein and Boes [89] is nothing but the subsequent study of Wettstein and Boes [88], with a clear emphasis on the social preferences in negotiations. This is the reason why it has not been included as a study on its own in this systematic review. The main result is that most participants clearly look at the patient’s incremental survival expectancy.

20 Patients are categorized into passive patients, who cannot select the physician and active ones who select the clinician providing the highest number of medical services.

21 In the medical framing, the allocator is a physician and the recipient is a patient.

22 Alhert et al. (2013)’s design contains some modifications with respect to Alhert et al. (2012): the number of potential recipients which decreases from 7 to a maximum of 3, different payoff components and new allocations offered with consequent new types of individuals.

23 Medical students’ altruism is also measured in a sender-receiver deception game by Besancenot and Vranceanu [8]. The paper is not included in the review given its neutral framing.

24 In the field, clinicians’ adherence to the protocols during patients’ consultations is measured. There, a positive correlation between clinicians’ generosity and the quality of provided care is found.

25 In the cost dispersion games, the third-party payer takes the role of the decision-maker.

26 Choices are presented as a budget line where each point stands for a different allocation [32].

27 Equality-efficiency trade-off stands for the tension between reducing differences in payoffs and increasing the total payoff.

28 Notice that Li [64] uses data from the experiment by Li et al. [63]. For this reason, it is not included in the current review.

29 Notice that one of treatment is always more patient-oriented than the other one.

30 Under the negligence rule, the physician must compensate the patient only if he has demonstrated negligence in at least one consultation.

31 Notice that further details of the protocol have been previously published in Saposnik et al. [79]. For this reason, since Saposnik et al. [79] and Saposnik et al. [80] are based on the same experiments, surveys, and simulated cases, we decided to include only one of the two above-mentioned papers in this systematic review.

32 Differently from Sect. "The role of payment schemes", given the wide variety of topics covered throughout the paragraph, it would be uninformative to the reader to compare experimental results with theoretical predictions. In general, we can say that the theoretical background is certainly crucial in any of the issues considered, but it is also important to investigate how theory is translated into evidence, that is where experimental economics comes into play, allowing to combine different scenarios which are instead difficult to replicate or simply to follow in the field.
